# Challenges of mismatching timescales in longitudinal studies of collective behaviour

**DOI:** 10.1098/rstb.2022.0064

**Published:** 2023-04-10

**Authors:** Mina Ogino, Eli D. Strauss, Damien R. Farine

**Affiliations:** ^1^ Department of Evolutionary and Environmental Studies, University of Zurich, Winterthurerstrasse 190, 8057 Zurich, Switzerland; ^2^ Department of Collective Behaviour, Max Planck Institute of Animal Behavior, Am Obstberg 1, 78315 Radolfzell, Germany; ^3^ Centre for the Advanced Study of Collective Behaviour, University of Konstanz, Universitatsstrasse 10, 78464 Konstanz, Germany; ^4^ Department of Integrative Biology, Michigan State University, 104 Natural Science Building, East Lansing, MI 48824-1115, East Lansing, MI 48824, USA; ^5^ Division of Ecology and Evolution, Research School of Biology, Australian National University, 46 Sullivans Creek Road, Canberra, ACT 2600, Australia

**Keywords:** collective behaviour, community detection, demographic, GPS tracking, long-term data, social network analysis

## Abstract

How individuals’ prior experience and population evolutionary history shape emergent patterns in animal collectives remains a major gap in the study of collective behaviour. One reason for this is that the processes that can shape individual contributions to collective actions can happen over very different timescales from each other and from the collective actions themselves, resulting in mismatched timescales. For example, a preference to move towards a specific patch might arise from phenotype, memory or physiological state. Although providing critical context to collective actions, bridging different timescales remains conceptually and methodologically challenging. Here, we briefly outline some of these challenges, and discuss existing approaches that have already generated insights into the factors shaping individual contributions in animal collectives. We then explore a case study of mismatching timescales—defining relevant group membership—by combining fine-scaled GPS tracking data and daily field census data from a wild population of vulturine guineafowl (*Acryllium vulturinum*). We show that applying different temporal definitions can produce different assignments of individuals into groups. These assignments can then have consequences when determining individuals' social history, and thus the conclusions we might draw on the impacts of the social environment on collective actions.

This article is part of a discussion meeting issue ‘Collective behaviour through time’.

## 1. Introduction

At the heart of any group decision are the conflicts of interest among individuals within the collective. When the preferences of individuals do not align, the collective must either resolve conflicts of interest through consensus (e.g. to move in a specific direction or to change from one behavioural state to another) or choose not to act as a collective (e.g. group fission [1]). Theoretical and empirical studies have focused on how consensus can be reached through democratic decisions [2,3], how costs of reaching consensus can cause collectives to fission [4,5] and how variation among individuals within groups shapes collective behaviour [6–9]. By contrast, less is known about how differences in preferences among individuals arise to produce the conflicts of interest that stimulate the need for consensus in the first place [10]. One challenge to understanding the origins of conflicting interests within collectives is that preferences are the outcome of processes that take place over a range of timescales. While integrating over time is a challenge for many fields (e.g. mate choice [11], dominance [12], social learning [13]), here we argue that it also merits attention in the field of collective behaviour. This is because collective actions emerge from interactions among many individuals, which can mask the distinct experience-driven preferences of each contributor.

**Figure 1.**
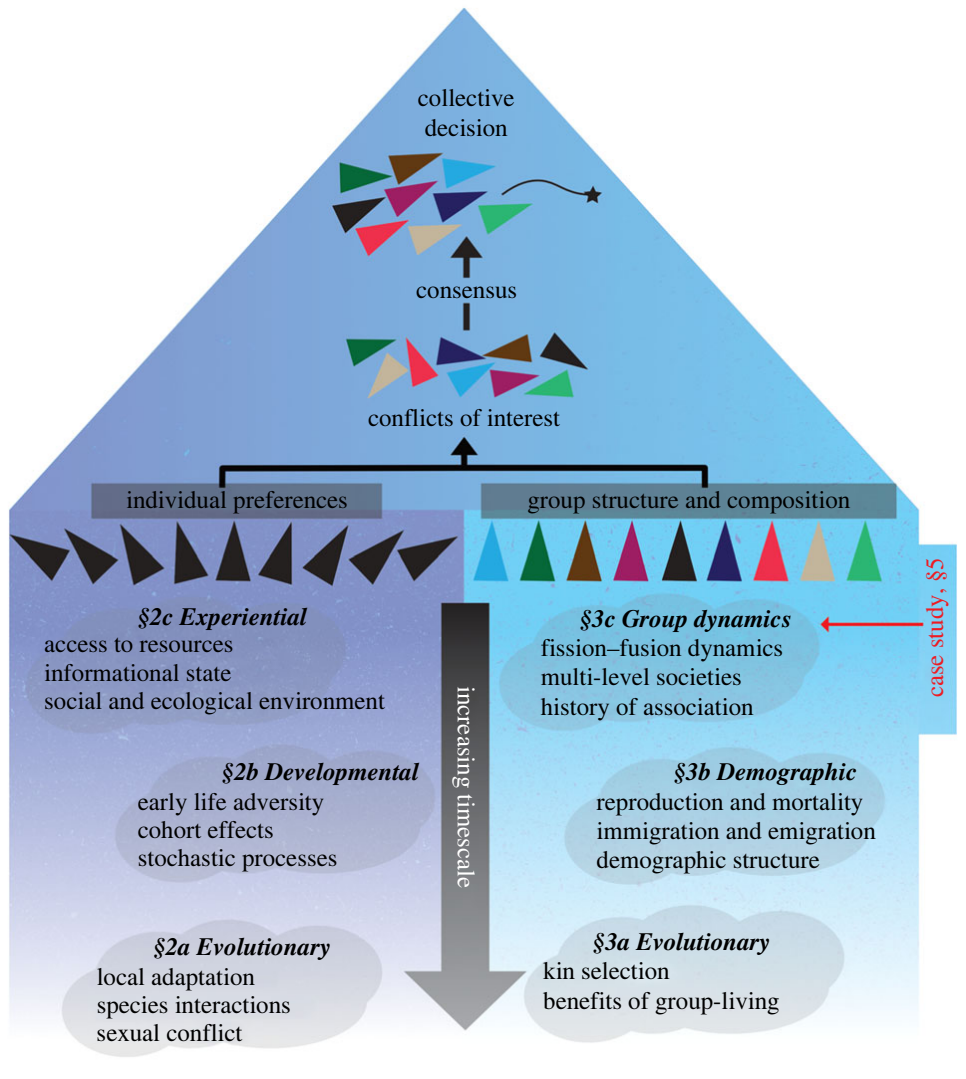
Overview of processes at different timescales shaping moment-by-moment collective behaviour. Two processes (group structure and composition, and individual preferences) lead to conflicts of interests among individuals in a collective, which requires the collective to reach consensus, then finally the collective can perform collective behaviour. We review each process in subsequent sections in this article and illustrate the methodological challenges in the case study. (Online version in colour.)

To guide research into collective behaviour, we review processes acting at multiple timescales to produce the conflicts of interest that prompt consensus decision-making (figure 1). These processes fall into two categories—individual-level processes and group-level processes. First, drivers of individual preferences shape the degree to which individuals have divergent interests, and thus the magnitude of consensus costs borne by members of the collective when engaging in collective behaviour [4,10]. Second, processes influencing groups determine which individuals—and consequently which preferences—are present in the collective, and shape the structure of the societies in which collective decisions are made. We then illustrate the methodological challenge associated with considering individual- and group-level processes operating at multiple timescales. Specifically, we present a case study showing how applying different time frames when defining groups can produce different assignments of individuals into groups (figure 1). We highlight how these differences might arise from methodological trade-offs or choices, that different definitions of group membership can generate different estimations of key properties of the social environment that individuals experience (e.g. group size, social stability, group dispersion), and that the properties of groups might capture social processes that occur at different timescales (e.g. arising from who is currently present versus historical group membership). Our case study, therefore, shows the conceptual challenge that arises when attempting to link even the most fundamental property of group-living to the collective actions that are expressed by a group.

## 2. Processes influencing individual preferences

Conflicts of interest arise when individuals have different preferences about how the group should behave. The drivers of individual differences in preferences can arise over a range of timescales. Here, we briefly survey the processes shaping individual preferences at evolutionary, developmental and experiential timescales.

### (a) Evolutionary timescale

Local adaptation, interspecies interactions and sex-differences shape the degree to which individuals within collectives have divergent preferences. Selection imposed by historical ecological conditions can lead individuals that are adapted to different environments to have divergent preferences. For instance, exposure to different predation regimes can shape individual preferences during collective movement [14,15]. If groups include individuals adapted to different local foraging regimes, these groups may experience higher consensus costs [10] when making foraging decisions than groups who are locally adapted to share the same preferences. Similarly, heterospecific groups need to reconcile species-differences in foraging preferences to remain as a cohesive unit [16–18]. Differences in preferences between the sexes can also produce conflicts of interest during collective behaviour. This could arise from sexual dimorphism in size or gait [19]. For instance, sex-specific preferences for activity budgets in red deer (*Cervus elaphus*) drive intersexual segregation [20,21]. Additionally, sexual conflict is a special case of sex-based conflicts of interest, where optimal reproductive strategies for males and females entail different collective behaviours. For instance, male banded mongooses (*Mungos mungo*) pay the cost of intergroup encounters, while females benefit from these encounters by mating with extragroup males [22]. Consequently, females are more often the initiators of intergroup encounters producing high consensus costs for males [23].

### (b) Developmental timescale

Early life conditions are well known to impact the behavioural patterns of individuals [24–28]. Several studies on zebra finches (*Taeniopygia guttata*) have demonstrated that early life adversity can impact social preferences among individuals, with individuals experiencing stress being less selective in their social interactions [29,30] and in which individuals they socially learn from (and therefore what information they obtain) [31]. Cohort effects, where cohorts of individuals differ from each other as a result of some shared early life conditions [11,32], can lead to sets of individuals that share the same preferences but differ from other sets of individuals. For instance, individuals experiencing the same early life environments may share preferences for activity budgets, movement patterns or foraging strategies [33]. Conflicts of interest could, therefore, also arise when these individuals disperse and mix with other cohorts to form groups [34]. Even among individuals raised in the same cohort and under the same conditions, stochasticity during their development can shape individuals' behavioural patterns later in their lives. For example, clonal fish (Amazon molly, *Poecilia formosa*) express consistent individual differences in their movement behaviour, even when reared under nearly identical social and physical conditions [35,36]. In sum, the extent to which individuals within a group have conflicting interests depends upon their individual and shared developmental histories.

### (c) Experiential timescale

Recent experiences also shape the preferences of individuals involved in collective decision-making. For example, accessing resources influences individual nutritional states and informational states about where future resources can be found, both of which can have strong effects on group structure [37], movement [38] and aggregation [39]. Intragroup variation in access to resources can introduce conflicts of interest about what a group should do next. When foraging on a patch of resources, vulturine guineafowl (*Acryllium vulturinum*) that are excluded from the patch are motivated to continue searching for new food patches, causing the group to initiate movement and forcing those that are still feeding to leave the patch to follow the group [40]. Experiences with the social and ecological environment are also a key component shaping behaviour. For instance, individual experiences in foraging ants influence behavioural specialization and division of labour within the group [41]. Variation in information among individuals can arise from differences in age (and thus different amounts of experience) or from the ‘passenger effect’, where followers cannot recall routes as effectively as leaders [42]. The role of differences in knowledge about the environment in shaping collective actions is exemplified by older elephants (*Loxodonta africana*) [43] and killer whales (*Orcinus orca*) [44] which lead their groups to rarely used resources during periods of food scarcity.

## 3. Processes influencing group composition and structure

Group composition and structure influence collective behaviour by shaping conflicts of interest within the collective. The composition of a group determines the set of individuals that can potentially engage in collective behaviour together, whereas group structure shapes the extent to which interests are aligned. Here, we review processes that can introduce stochasticity in group structure and composition at evolutionary, demographic and dynamic timescales, thereby shaping conflicts of interest within collectives.

### (a) Evolutionary timescale

The evolution of social systems—the mating system, social organization and social structure [45]—shapes the potential for conflicts of interest within collectives. Patterns of dispersal and mating influence the kinship structure of groups [46], which in turn impacts the extent to which individuals within the group have aligned interests [47]. For example, when conflicts of interest among kin occur, consensus costs are offset by the inclusive-fitness benefits of pursuing the interests of related group-mates [48]. Finally, the selective pressures leading to the evolution of aggregations of individuals influence the relevant domains in which conflicts of interest might occur. For example, forming and maintaining groups can benefit individuals by increasing their ability to detect predators [49], increasing their ability to find food [50], improving their navigation ability [51–53] or reducing the energetic costs of movements [54]. However, differences in preferences can arise across all of these domains, including whether risk is present [55], what food patches to choose [56], which direction to move in [2] or the speed of locomotion [19]. These domains can also intersect. In wildebeest, synchronous birthing has co-evolved with large-scale migration [57], creating a need for consensus both in birth timing and when and where to migrate.

### (b) Demographic timescale

Demographic processes shape conflicts of interest within groups by influencing the composition [6], social network structure [58] and stability of social groups [12]. The addition and subtraction of individuals through births, deaths, immigration and emigration shape group size and structure, which can impact the expression of collective behaviours [59,60]. For instance, the addition of uninformed individuals to a group increases the likelihood of the group deciding in favour of the preferences of the majority [3], while demographic turnover (the introduction of naive individuals) can increase the rate at which cultural change takes place in animal groups or populations [61]. When the results of prior collective behaviour influence future collective action, for example via the memory of some group members [62], then historical group composition can impact future behaviours. Demographic turnover can, therefore, influence the maintenance and efficacy of collective strategies [61,63].

### (c) Group dynamics timescale

Collective behaviour is also influenced by social dynamics within groups, like temporary splitting and joining of individuals or groups. For instance, groups characterized by high degrees of fission–fusion dynamics experience frequent changes in group composition [64,65], and social instability introduced by changes in group membership can impede collective behaviour. For instance, in captive zebra finches, changes in group composition reduced group foraging efficiency [66]. Group dynamics can be extrinsically driven, such as in prides of lions (*Pantera leo*), where the patterns of fission–fusion and the stability of membership into subgroups is affected by ecological conditions and the corresponding availability of prey [67]. Group dynamics can also be internally driven, such as when dominant vulturine guineafowl exclude subordinates from food patches, causing the latter to depart [40]. Group dynamics may also emerge at multiple levels of social organization. For example, in multi-level societies, where cohesive groups join with other groups to form higher-order groupings [68], consensus decisions may be influenced by both core group composition and the conflicts of interest that emerge from the higher-order groups. Finally, consensus and cooperation may be influenced by prior patterns of association at these different levels as well (e.g. familiarity, social bonds), such that long-term grouping patterns shape consensus during collective action. For instance, in the fission–fusion societies of spotted hyaenas, collective mobbing of lions is promoted by the presence of preferred subgrouping partners [69].

## 4. The challenge of mismatching scales in longitudinal studies of collective behaviour, and some solutions

The processes highlighted above point to a fundamental challenge in explaining collective behaviour: consensus and collective action are reached through fine-scale moment-by-moment interactions that resolve conflicts of interest, but these conflicts of interest originate in longitudinal processes that require us to reach into the past. This generates both methodological and conceptual challenges. On the one hand, groups have to be observed over longitudinal timescales that are relevant to establishing conflicts of interest, because the source of the conflicts of interest are rooted in the individual's and/or collective's history. On the other hand, very-high-resolution (cross-section of the longitudinal processes at the moment of the focal collective action, e.g. second-by-second) data are required on the relative movements of many individuals to establish how preferences are integrated into collective actions [2,70]. Additionally, collectives have to make different types of decisions (e.g. when to move versus where to move), and these likely represent different axes of decision-making [71], which could be more sensitive to some timescales than others. For example, what a group does next might be influenced by the distribution of present nutritional states within a group, whereas where a group goes next may be influenced by the longer-term historical membership of the group and what the present individuals learnt from them. Embedding collective behaviour research in long-term individual-based study systems offers an approach to tackling these challenges, by allowing the collection of new fine-scale data on consensus formation that is informed by rich longitudinal information on individual and group histories over multiple timescales.

Several shorter-term approaches have also helped shed light on the links between longitudinal processes and collective behaviour. Three, in particular, have substantial potential for continued insight—mixed-species collectives, artificial selection experiments and the use of clonal species. Mixed-species collectives provide interesting opportunities to understand the role of direct benefits in the evolution of social and collective behaviours, as by definition individuals cannot gain indirect fitness from cooperating with a heterospecific [72]. Such studies have demonstrated how individual social rules appear to be tuned differently to conspecifics versus heterospecifics [56,73], which provides a potentially powerful experimental paradigm in systems where groups can be experimentally generated [74]. Recent work with guppies (*Poecilia reticulata*) has also demonstrated the potential for using within-species variation to understand the evolutionary dynamics of collective behaviours, specifically the timeframe over which alignment can evolve [75]. Finally, clonal species provide an intriguing opportunity to understand how individual differences emerging from developmental conditions could affect the expression of, or individual contributions to, and performance and consensus costs, of collective actions [76]. For example, tests of collective behaviour in experimental groups where clonal individuals experienced homogeneous versus heterogeneous developmental conditions offer a promising approach for linking processes taking place at different timescales.

Recent technological improvements are now facilitating three important GPS-based approaches that provide another promising avenue for unpacking the multiple timescales influencing collective behaviour—continuous GPS tracking, lifetime GPS tracking and whole-group tracking. With solar-power technology, it is becoming increasingly feasible to study not only the choices that individuals make, but also the consequences of these choices on future decisions. In group-living species that maintain high cohesion, this can even be achieved by tracking just a few group members [70]. Further, it is becoming increasingly feasible to capture, in detail, both the physical [77] and the social [70] environments that individuals experience over their lifetimes. Such approaches become particularly powerful when combined with whole-group tracking, allowing the relative contributions of each individual to be studied over time [2,78]. In such studies, demographic changes, such as deaths of individuals or immigration by others, provide powerful natural experiments that can shed light on how prior experience affects individual contributions to collective actions. Finally, GPS data can be used to quantify spatial aspects of the structure of groups, such as how cohesive they are or how efficiently they move.

In the first part of this paper, we reviewed different processes that can shape the preferences of individual and the structure of societies, and how these processes can operate at different timescales from the collective behaviours that they shape. However, while our methods to study animal collectives have improved, we must still overcome a number of methodological and conceptual challenges. One particular challenge that spans both methodological and conceptual dimensions is identifying the correct time frame at which a given hypothesized driver operates to shape preferences and the behaviour of collectives. This includes one of the most foundational concepts for collective behaviour: what is a group? From a conceptual perspective, there is the challenge of distinguishing whether a collective behaviour is being shaped only by the individuals present or whether there are legacy effects from past group members. Examples of this include culturally transmitted behaviours, which can lead to between-group differences in behaviour arising not from current group members but from their predecessors in which the behaviour first arose [79]. From a methodological perspective, there exists a trade-off between applying finer-scale definitions (e.g. moment-by-moment group membership) and uncertainty in the estimates of which individuals are present in the group (or *vice-versa*, uncertainty about which group an individual belonged to over a longer time period). In the following case study, we illustrate the concept of mismatching timescales by applying different temporal (and methodological) definitions of social units. We then demonstrate how these different definitions translate to different estimates of group properties, thereby introducing uncertainty in downstream analyses.

## 5. A case study of mismatching timescales: defining the membership of social units in vulturine guineafowl (*Acryllium vulturinum*)

Defining groups (herein *social units*) is often the first step to investigating collective decision-making. However, social environments individuals experience change constantly (see §1), which can make operationalizing the definition of social units less straightforward. Depending on the time window we chose, the social units we detect (membership and composition of supergroups, groups and subgroups, temporal fission from a group, etc.) can differ. This will then have consequences for our estimations of how the membership and composition of social units shapes collective behaviour (e.g. which individuals' preferences might form part of a given decision). Thus, defining social units requires addressing two questions: (i) What level of social units is meaningful when interpreting a given current collective action? and (ii) How can we choose the corresponding timescales to define the focal social units?

For this case study, we assess how membership dynamics and structure vary over three different timescales, in a multi-level society of vulturine guineafowl where social units do not have any central resources to regularly come back to (e.g. colonial nesting locations) and often mix with other groups in space and time. We define social units as sets of individuals that are inferred to maintain cohesion across time, which is consistent with existing definitions: sets of individuals that maintain close spatial proximity (mean intra-unit distance is substantially less than the distance over which individuals range daily, and smaller than mean inter-unit distance) over days, and among which most social interactions occur (following [80,81]).

### (a) Study system

The vulturine guineafowl project has been collecting long-term GPS tracking and daily census observations of aggregations (field-observed groups) in a wild vulturine guineafowl population at the Mpala Research Centre in Kenya. Vulturine guineafowl are predominantly terrestrial and highly gregarious, living in a multi-level society [82] in which individuals are regularly observed with up to 100 (or sometimes more) conspecifics. Individuals purportedly belong to the same social unit over multiple years or seasons, with these members moving highly cohesively [82]. However, a social unit can also temporarily split into subunits for a few hours up to several weeks, eventually merging back together to re-form the original single social unit. Social units can also form supergroups that can last for a few days up to several months (especially during dry seasons) that then disband back into the original social units. These dynamics, combined with sporadic observational data, can make it challenging to determine exactly what social environments an individual might have experienced for a given study period.

### (b) Data collection

Approximately 90% of individuals in the study population are uniquely identifiable in the field based on a unique combination of colour bands fitted to their legs (*n* = 782 individuals during the study period). We collect daily census data (morning and evening), during which we record observations of individuals moving and associating together. However, we do not observe all individuals every day (or sometimes week), meaning that estimating social units at finer timescales (e.g. within a month) can result in having fewer observations of individuals and, correspondingly, greater uncertainty in the assignment of individuals into social units.

When encountering an aggregation, we record the number of marked and unmarked individuals and the identity of all individuals present. We also record whether the observed sets of individuals (clusters of individuals found to be closer to one another than to others) behave as a cohesive unit (‘single’ set of individuals), or as multiple units that arrived from or are moving in different directions (‘multiple’ sets of individuals). For the purpose of this study, we used only ‘single’ observations for the data analysis. Further, we removed incomplete daily census with any missing information to reduce uncertainty of estimated networks [83]. We removed individuals that were observed two or fewer times in a focal period, because detected social networks are unreliable when the number of observations is very small [83], although the methods below are relatively robust to undersampling [84]. For this study, we use data collected across eight continuous months (1 September 2020 to 30 April 2021), with varying intensity of data collection due to periods when birds temporarily left the study area [85].

To generate measures of social unit properties (see §5e), we used GPS data from 66 males fitted with solar-powered GPS tags (e-obs 15 g solar). GPS tags were programmed to collect a mixture of high-resolution data. When the battery charge is high, tags collect 1 Hz bursts of data sometimes lasting several hours. When the battery charge is lower, tags collect a burst of 10 consecutive points every 5 min. See [70] for more details on the design of the GPS study. For the purpose of this case study, we subsampled any 1 Hz data to one point every 5 min. We used GPS data from 1 October 2020 to 31 March 2021.

### (c) Methods: inferring social units over multiple timescales

**Figure 2.**
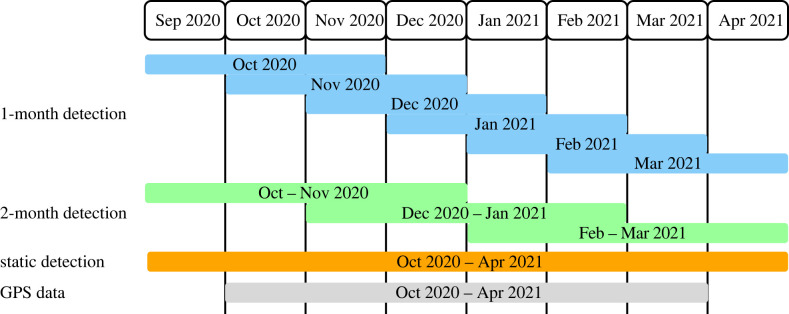
Summary of focal periods for each detection period. Census data from September 2020 to April 2021 was used to determine group membership. For *1-month* and *2-month* detections, the census data from 1 month before and after the focal month(s) were used to detect group memberships. For *static* detection, all census data used for *1-month* and *2-month* detections were used. We used GPS data only from October 2020 to March 2021, because our aim was to measure cohesiveness of detected groups within focal months. Blue, green and orange represent *1*-*month*, *2-month* and *static* detection, respectively, throughout this paper. (Online version in colour.)

Since the main aim of this case study is to show whether and to what extent social unit structure and membership emerge over multiple timescales, we specifically report the outcomes from eight different approaches to inferring social units from census data (table 1 and figure 2). These vary in terms of the time frame (1-month, 2-month, 8-month), whether we employ a bootstrapping procedure to better account for low sampling at finer timescales, and whether we use a dynamic network community algorithm to track the carryover of membership to social units across time. We expect to observe differences in the allocation of individuals to social units depending on time frame applied. From observations, we know that a vulturine guineafowl group sometimes temporary splits as subunits consisted of non-repeatable members, and that multiple groups can be observed together at the overlapping parts of their home ranges, with these dynamics occurring on a daily basis. Some of these dynamics also vary over longer time frames, for example groups merging to form a supergroup under harsh ecological conditions, such as during droughts. We can, therefore, expect the shorter time frame to capture temporal or daily dynamics of day-to-day association patterns, while the longer time frame can capture more general patterns of group memberships.

**Table 1.** Summary of approaches for inferring social units. For 8-month approaches, the detection of carryover membership was not applicable (n.a.) because these have only one sampling period.

approach	sampling period	outcome network	bootstrap	carryover membership
1-month^§^	3 months**	1-month basis	yes	no
1-month	3 months**	1-month basis	no	no
1-month*^§^	3 months**	1-month basis	yes	yes
1-month*	3 months**	1-month basis	no	yes
2-month^§^	4 months**	2-month basis	yes	no
2-month	4 months**	2-month basis	no	no
2-month*^§^	4 months**	2-month basis	yes	yes
2-month*	4 months**	2-month basis	no	yes
8-month^§^	8 months	8-month basis	yes	n.a.
8-month	8 months	8-month basis	no	n.a.

*Approach used detection of carryover of community membership across 1 or 2 months.

**Data for each month includes data from the months before and after, using a sliding window method (figure 2).

^§^Approach used bootstrapping.

For 1-month basis analyses (1-*month*, 1-month*) and 2-month basis analysis (2-month*), we aggregated census data from 1 month before through to 1 month after the focal period as a moving window, resulting in six 1-month social networks and three 2-month social networks (figure 2). For the static network (*8-month*), we combined all census data for the entire study duration (8 months) to create one social network.

To create each network, we first created a group-by-individual matrix in which each cell contained a 0 or 1 representing whether each individual (columns) was observed in each aggregation (row) that was encountered during that focal period. We then calculated the network for the focal period using the get_network function in the *asnipe* package [86] in R [87]. Ties in these networks were calculated as the simple ratio index of association, which estimates the proportion of time that two individuals were together by dividing the number of census observations of them together by the number of possible chances that they could be observed together [88]. When the edge weights (range 0 = always apart to 1 = always together) were less than 0.5, we replaced these by 0 because community detection algorithms (see below) are substantially more sensitive to the presence of an edge than they are to the edge weights.

Next, we inferred social communities from the social network as a means of extracting social units. Community detection algorithms detect sets of individuals that are more connected among each other than they are to other individuals. We did this in two ways, either directly on the observed network for each focal period, or using a bootstrapped meta-network approach for each focal period to better account for uncertainty at finer timescales. For community detection (on both types of network), we used the walktrap community algorithm in the R package *igraph* [89], which was previously found to perform best with census observations of multi-level societies [90]. The meta-network approach consists of using a bootstrapping approach following the algorithm described by Shizuka & Farine [84]. Briefly, the approach involves resampling the observed observations of aggregations with replacement (i.e. bootstrapping), constructing the network and detecting the membership of individuals to communities in the network. We could calculate the probability of observing two individuals in the same community across all the bootstrapped replicates (*n* = 100), which we defined as our meta-network. We then ran the walktrap community algorithm on this meta-network to get the community membership for the focal period.

Because of the tendency for community detection algorithms to include complete connected components in sparse networks, we added an extra checking step in the meta-networks. We first checked the size of the detected communities and compared these with the maximal number of individuals with colour bands observed in a ‘single’ census observation during the focal period. When the size of a detected community was higher than the highest number of individuals encountered in a field observation during the same period, we re-ran the procedure above after subsetting the census observation data to include only the individuals assigned to that detected community. This procedure allows us to partition observed aggregation of multiple groups into their composing social units.

### (d) Methods: detecting carryover membership of social units across focal periods

We also used a dynamic network community algorithm to link the community membership across time for the 1-month and 2-month networks (table 1). We used the MajorTrack library [91] in Python, which produces global community identifiers, and therefore links the community identifiers across consecutive time periods. In some cases, the community remained stable but some individuals were missing in a given time period, so we also used interpolation to re-add temporarily missing individuals into their community.

### (e) Methods: measuring cohesiveness and dynamics of detected social units

To quantify how group cohesiveness emerges over the different timescales at which we defined social units, we used the GPS data to estimate the average GPS pairwise distances of individuals over each focal period. These data give us insights into our ability to capture social units that are highly cohesive in their movements versus social units that represent longer patterns of (re-)associations. We did this by calculating the mean and maximum daily GPS pairwise distances among individuals within the same detected community. We used GPS data only from males, which are philopatric [92], as the few dispersing females in our dataset could substantially impact the estimates of pairwise distances.

To quantify the temporal stability of group membership inferred when using different timescales, we calculated Jaccard similarity between the detected social network communities in consecutive focal periods. This is calculated as the ratio of (i) the number of individuals detected in the same community across sequential focal periods (e.g. in month *N* and month *N* + 1) to (ii) the number of unique individuals detected in the community during either focal period (e.g. in month *N* or month *N* + 1). Note that we could only do this for approaches that used the carryover methods as these provided the necessary information about the links between communities over time.

### (f) Methods: quantifying effects from different methodological procedures on detected social units

Finally, we quantified how methodological processes applied in different approaches can explain differences in cohesiveness of detected communities. Specifically, we used generalized linear models with fitting the size of detected communities (Poisson) or average GPS pairwise distance (lognormal) as a response variable and fitting time window (*1-month*, *2-month*, *8-month*), bootstrapping process (yes/no) and detection of carryover membership (yes/no) as predictors, using the *lme4* package [93] in R [87]. To test the effect on group stability, we used generalized linear mixed models (binomial) with fitting the similarity of group memberships as a response variable, fitting time window (*1*-*month*, *2-month*, *8-month*), bootstrapping process (yes/no) and detection of carryover membership (yes/no) as fixed effects, and fitting sampling period ID as a random effect. To test the effect from each fixed effect, we used a two-way ANOVA (type 3), in the *car* package [94] in R [87].

### (g) Results

**Figure 3.**
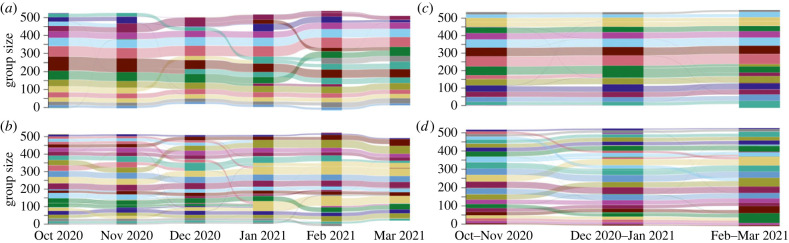
Detected network community dynamics over time using carryover methods. (*a*) 1-month*^§^: 1-month detection with carryovers of community membership and bootstrapping. (*b*) *1*-*month* *: 1-month detection with carryovers of community membership without bootstrapping. (*c*) 2-month*^§^: 2-month detection with carryovers of community membership and bootstrapping. (*d*) *2-month* *: 2-month detection with carryovers of community membership without bootstrapping. Each colour represents an inferred social unit, and colours were randomly assigned within each approach. The shaded colour shows the track of social units between focal periods, and the movement of individuals between social units. The colour changes when a social unit merged with another social unit or split into smaller subunits. The height of each colour bar corresponds to the number of individuals in the social unit. (Online version in colour.)

With bootstrapping, we detected 98 (15–19 per focal period), 21 (14–18), 50 (15–18), 21 (16–18) and 16 unique social units when using the *1-month*^§^, 1-month*^§^, *2-month*^§^, 2-month*^§^ and *8-month*^§^ approaches, respectively (figure 3). Note that approaches with detection of carryover memberships (*) often have the same social unit IDs across focal periods, thereby reducing the total number of inferred social units. In total, 571, 571, 568, 572 and 572 individuals were included in the detected communities for each approach, respectively (one individual never had sufficient observations at a 1-month timescale to be included in the dataset). Without bootstrapping, we detected 148 (22–27 per focal period), 44 (22–27), 77 (24–29), 32 (24–26) and 34 unique groups by *1*-*month*, *1*-month*, *2-month*, 2-month* and *8-month* approaches, respectively (figure 3). In total, 567, 567, 560, 564 and 531 individuals were included in the detected social units for the non-boostrapped approaches, respectively. Applying the bootstrapping procedure had a significant impact on the estimated size of social units (*β* ± s.e. = 0.474 ± 0.014, *χ*^2^ = 1085.54, *p* < 0.001; electronic supplementary material, table S1).

**Figure 4.**
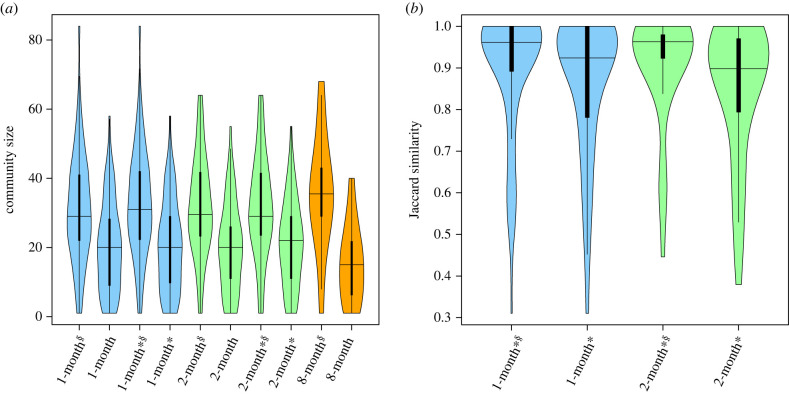
The distribution of detected social unit sizes and Jaccard similarity between social units detected consecutively. (*a*) The distribution of social unit sizes inferred from each method. The *x*-axis shows each community detection method (dynamic community detection with and without bootstrapping, static community detection), and the *y*-axis shows the size of detected community. (*b*) Jaccard similarity for communities in consecutive focal periods when using the dynamic network community method. *Approach using detection of carryover of community membership across periods of 1 or 2 months. ^§^Approach using bootstrapped meta-networks. Note that we could not calculate Jaccard similarity for approaches without the dynamic network community method as there was no way to link networks in consecutive focal periods. (Online version in colour.)

Inferred social unit size varied depending on the community detection approaches (figure 4). Social unit sizes from the shorter-term approaches (1-month, 2-month) tended to be smaller (within a focal period) than the 8-month network when using bootstrapping. Social units were substantially larger (and less variable) when using the bootstrapped meta-networks than when using only the observed network. Further, the bootstrap procedure produced substantially fewer unrealistic social units that consisted of only a few individuals (figure 4). Overall, social units detected by the 1-month detection approach and the 2-month detection approach had similar Jaccard similarities (figure 4). However, some carryover of community membership detected without the bootstrapping procedure experienced more turnovers in their community memberships (lower Jaccard similarities) than those detected with the bootstrapping procedure did (figure 4; *β* ± s.e. = 0.287 ± 0.069, *χ*^2^ = 17.47, *p* < 0.001; electronic supplementary material, table S2).

**Figure 5.**
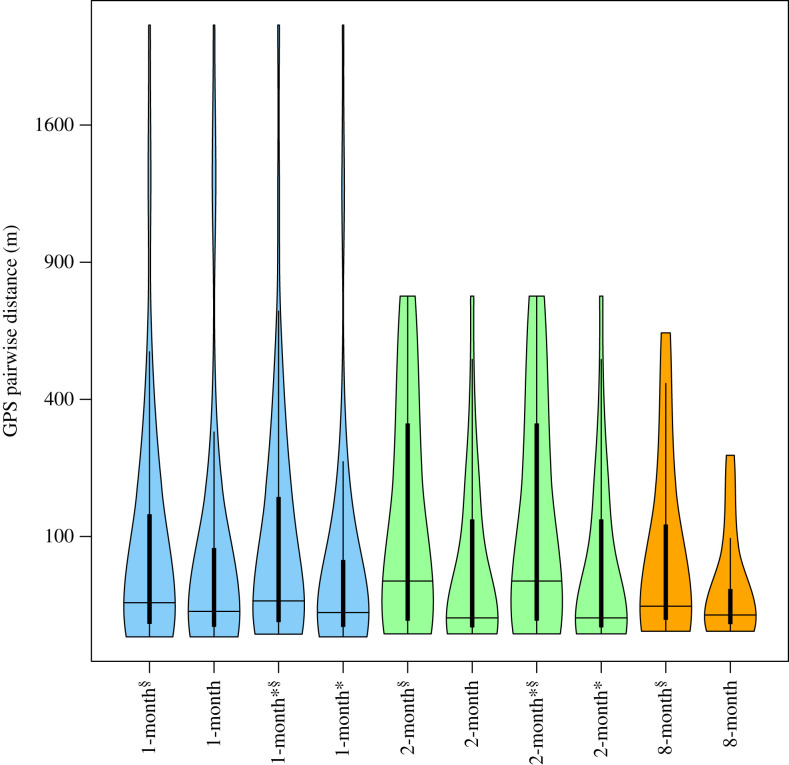
Averaged GPS pairwise distances within detected communities. The *x*-axis shows the community detection approach (table 1) and the *y*-axis shows GPS pairwise distance among tagged males within the same detected community (m). *Approach using carryover of community membership. ^§^Approach using bootstrapping. (Online version in colour.)

Estimated group cohesiveness varied depending on the community detection approach used (figure 5). Overall, the 2-month community detection approaches produced higher and more variable average GPS pairwise distances among individuals assigned to the same community than other approaches did. Groups detected without bootstrapping had substantially lower average GPS pairwise distances than the methods with bootstrapping (*β* ± s.e. = 0.479 ± 0.154, *χ*^2^ = 9.71, *p* = 0.002; electronic supplementary material, table S3). The 8-month detections (with the bootstrapping procedure) had substantially smaller and less variable average GPS pairwise distances, compared with other approaches (electronic supplementary material, table S3). Detection of carryover social units did not impact cohesiveness of detected groups in most approaches (but, without bootstrap, 1-month detection approaches produced higher and more variable groups without detection of carryover groups).

### (h) Summary of the case study

We used this case study to investigate the inference of social units in a multi-level society using different temporal and methodological approaches. The differences in timescales used, and whether or not methods were used to carry over community membership, led to substantially different inferred social unit sizes, stability and spatial cohesion. Community detection approaches without bootstrapping produced more communities with more variable community sizes, as well as more communities consisting of a few individuals, relative to methods using bootstrapping. Thus, analyses at shorter timescales may suffer from higher uncertainty in the allocation of individuals into social units. Because very small social unit sizes (i.e. single individuals) are unrealistic in vulturine guineafowl, we conclude that the bootstrapped meta-networks produce better representations of the social unit structure in the population when focusing on identifying social units at shorter timescales. However, bootstrapping produced social communities with higher average GPS pairwise distances among males within the same inferred social unit, relative to the non-bootstrapped approaches. In biological terms, this means that the detection approaches without carryover of community membership and without bootstrapping may be more sensitive to detecting fine-scale temporal changes in social unit membership (i.e. which individual is actually present at a given point in time). However, these changes may not be perceptibly different from inference errors. Across different timescales, shorter-term focal periods (1 month) typically produced the smallest pairwise GPS distances, and only slightly higher variability in the Jaccard similarity of social unit membership across time. This corresponds with more accurately capturing the social environment that individuals experienced during the focal period.

So which method is best? This depends, in large part, on the question, thereby highlighting the conceptual challenges that arise when defining social units. One objective measure would be a definition that minimizes GPS pairwise distance while maximizing the stability of the estimated groups. Here, we found that focal periods of 1 month produced relatively low GPS pairwise distances, high Jaccard indices and (when using bootstrapping) larger social unit sizes when guineafowl groups formed supergroups (according to field observation). This suggests that there may be an optimal timescale for inferring stable social units that are not susceptive to temporal day-to-day changes in associations but that still accurately capture demographic changes. The 1-month definition (1-month*, 1-month*^§^) was also the only one able capture larger-scale changes, such as those driven by ecological conditions. Previous studies and field observation have revealed that vulturine guineafowl form a multi-level society [82], and evidence is emerging that social units merge during dry seasons to form supergroups that consist of about 70–100 individuals (and, more recently, we have observed a supergroup with an estimated 600 individuals). However, while many questions will focus on the finer-scale collective actions, others may instead need to capture the most stable social units (e.g. studies of cultural transmission). We found that GPS pairwise distances for the 8-month detection were smaller than shorter-term detections with carryover memberships. This could point to more aggregated approaches as being better at capturing the most stable set of individuals that compose a social unit (i.e. stable across different environmental conditions).

Ultimately, our case study reveals some trade-offs between detecting stable and strong relationships between individuals and capturing the dynamics of aggregations over time. Longer time periods capture the former, but are unlikely to accurately describe what social environment an individual will have experienced in a given time and place. By contrast, using very short timescales makes it more challenging to track which individuals ultimately form a long-term social unit. Finally, bootstrapping methods (especially) were typically more inclusive, meaning that they were likely to allocate individuals to the same social unit even if they may have spent some time apart, whereas networks constructed directly from the observations were more likely to produce isolated individuals (and thus to separate individuals into unrealistic social memberships). Thus, depending on research questions, scientists need to carefully decide on which timescale and community construction approaches might produce meaningful social units for their research questions, all the while keeping in consideration how robust the method is at inferring social units. Through this case study we have not only provided some starting guidelines, but also demonstrated an analytical procedure that can help with choosing the best approach for a given question (i.e. optimizing the relative accuracy in terms of cohesiveness, social stability and/or social unit size).

## 6. Conclusion

Some behaviours can be affected by temporal surrounding social environments (e.g. which individuals are present at the moment), while others can be influenced by the longer-term or the broader social environments they have been experiencing across different timescales. Given such dynamics, when studying collective actions, what are the relevant definitions of groups? When individuals contribute to the decision-making process, which levels of social units have an influence? As we touched on in the case study, identifying meaningful social units, especially in societies where groups experience membership changes at both finer (e.g. subgrouping, demographic changes, dispersing) and larger scales (e.g. forming supergroups, splitting into multiple groups for a period of time) may not be straightforward because the membership inferred can depend on the timescale (and corresponding uncertainty) and the methods we choose to overcome limitations in the data. Studying social animals living in other types of societies also requires facing the same conundrum when drawing inference about the social structure that individuals experience at different timescales. For example, what levels of social units (e.g. surrounding associates at the moment versus the stable social group) shape current collective actions, when subsets of a group perform the collective action? Even in animal species forming closed societies without any fission–fusion dynamics between social units (such as territorial animal species), individuals' preferences for collective actions arise over various timescales (evolutionary, developmental, experiential), while groups continuously experience changes and development of collective actions under demographic timescales.

Once we detect the relevant social units (e.g. group memberships to be focused on), the next steps are finally to investigate how long-term effects shape moment-by-moment collective actions: do individuals decide based on the temporal surrounding social environments or the broader social environments they have been experiencing? Furthermore, how do these individual- and group-level dynamics impact collective actions at different spatial and social scales? For example, do demographic turnovers or dispersal events diffuse developmental effects over the landscape? As we discussed in §4 ‘The challenge of mismatching scales in longitudinal studies of collective behaviour, and some solutions’, conducting long-term comparative studies using clonal individuals or mixed species with a combination of the recent advancement of technologies could give further insights on these topics. We also believe that investigating these questions, with careful consideration of the discrepancy of timescales, can help researchers to develop a deeper understanding of how different long-term effects can shape current collective actions.

## Data Availability

All data and code to replicate our analyses are available at https://doi.org/10.6084/m9.figshare.21591153.v1.
